# Sigmoid perforation caused by an ingested chicken bone presenting as right iliac fossa pain mimicking appendicitis: a case report

**DOI:** 10.4076/1752-1947-3-7385

**Published:** 2009-07-31

**Authors:** Sandeep Joglekar, Iqbal Rajput, Sachin Kamat, Sarah Downey

**Affiliations:** 1 James Paget University Hospital, Lowestoft Rd, Gorleston, Great Yarmouth, NR31 6LA, UK

## Abstract

**Introduction:**

Gastrointestinal perforation due to a foreign body is not unknown. The foreign body often mimics another cause of acute abdomen and requires emergency surgical intervention. The majority of patients do not recall ingesting the foreign body. Perforations have been reported to occur in a pathologically abnormal colon.

**Case presentation:**

We report an interesting case of a 47-year-old Caucasian man who had a perforation of the sigmoid colon caused by an ingested chicken bone mimicking acute appendicitis. Our patient presented with right iliac fossa pain and local tenderness. When a laparotomy was performed, a chicken bone was found protruding through the sigmoid colon, which was found to lie in the right iliac fossa, thus mimicking acute appendicitis. Our case is different from previously reported cases in that perforation occurred in a non-pathological colon.

**Conclusion:**

Our case emphasises the fact that the operating surgeon has to be aware of various differential diagnostic possibilities which mimic acute appendicitis. This has implications on the training of junior surgeons who are often involved in performing these procedures, and may do so out of hours. Care needs to be taken while obtaining consent for the necessary operation.

## Introduction

Sigmoid colonic perforation is an acute surgical emergency, the most common cause of which is diverticular disease. Patients present with left iliac fossa pain, raised inflammatory markers and localised peritonitis. Traumatic perforation of the colon due to a foreign body affects the narrowest portion of the bowel, namely the ileocaecal or rectosigmoid junction. Earlier case reports of traumatic perforation of the sigmoid colon emphasise the presence of a background pathology such as diverticular disease, cancer or a fistula. Sigmoid mobility can result in pathologies and present as acute appendicitis. This clearly causes diagnostic confusion and can present a challenge especially to a trainee-grade surgeon.

## Case presentation

A 47-year-old Caucasian man presented with a 3-day history of colicky generalised abdominal pain, gradually getting worse, eventually localising to the right iliac fossa. The pain was continuous, getting aggravated by movement and relieved at rest. There was no nausea or vomiting. There were four episodes of watery diarrhoea without blood or mucus, which stopped after administering anti-diarrhoeal medication.

The patient had no past history of any medical or surgical problems. There were no known drug allergies, and he was not on any regular medications at the time. Clinical examination revealed pyrexia of 39°C, tachycardia of 104 per minute and normal blood pressure. Abdominal examination showed localised tenderness and guarding in the right iliac fossa. Laboratory studies revealed a white blood cell count of 16,400/mm^3^ with neutrophilia. C-reactive protein was at 318 mg/L. Urea, creatinine, electrolytes and liver function test results were normal. Dipstick examination of urine was normal. A provisional diagnosis of acute appendicitis was made, and consent was taken for appendicectomy. Because of the physical findings of localised peritonitis, a decision was made to perform a lower midline laparotomy.

**Figure 1:**
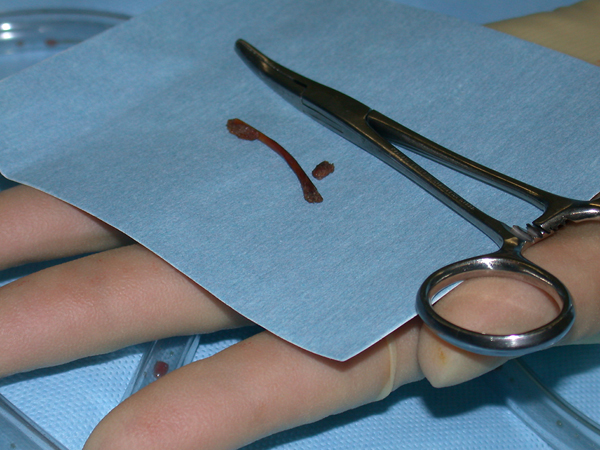
**Photograph showing the foreign body: a chicken bone**.

**Figure 2:**
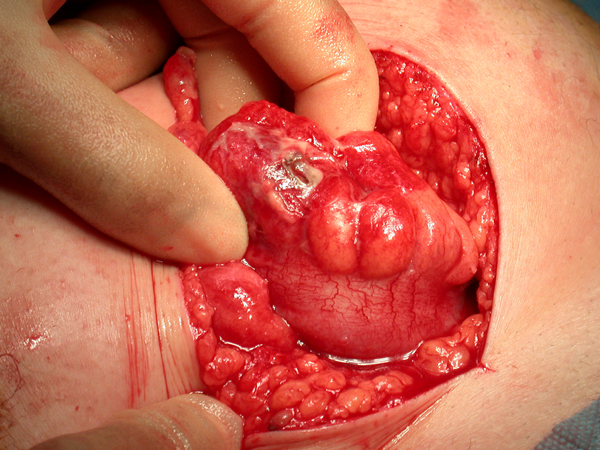
**Photograph showing the sigmoid colonic perforation**.

During the operation, a small (2 mm) perforation was found in the distal sigmoid colon through which a chicken bone was protruding outward (Figure 1). There was localised purulent collection, but no faecal contamination was found. The foreign body was removed and, after freshening the edges of the perforation, it was oversewn in two layers (Figure 2). The appendix was found to be normal and was left intact. The postoperative recovery was uneventful.

## Discussion

A variety of foreign bodies such as dentures, toothpicks, chicken bone, fish bone and cocktail sticks have been implicated in the pathogenesis of gastrointestinal perforation [1,2]. They are common in elderly individuals (who wear dentures), alcoholics, children and mentally retarded individuals. Fewer than 1% of ingested foreign bodies will perforate the bowel, and the greatest risk is with large, sharp or pointed objects. Although most sharp objects pass without complications, once beyond the oesophagus, they carry an increased risk of complications, including bowel obstruction, perforation and erosions into adjacent organs. Most commonly, the location of the perforation is the narrowest region of the bowel, either the ileocaecal valve or rectosigmoid junction [3,4]. There are reports of cases with a fulminant clinical course due to a fatal hepatic abscess [5].

Patients do not often remember having ingested the implicated foreign body, which is found either during radiological investigations or during surgery. Patients may have varied symptoms such as abdominal pain, nausea, vomiting, fever, haematochezia or melena. Foreign-body perforation of the bowel presents as acute abdominal emergency and often mimics conditions such as acute appendicitis, diverticulitis or perforated peptic ulcer. Free gas is not a common feature on the abdominal X-ray and was present in only 20% of patients [6,7]. One review of cases of a sigmoid colonic perforation due to a chicken bone found three main presentations: 1) colovesical fistula; 2) acute abdomen; and 3) inflammatory mass [8]. Two other reports revealed incidentally discovered sigmoid carcinoma after perforation caused by an ingested chicken bone [9,10]. Our patient presented with the classical symptoms of acute appendicitis as generalised abdominal pain with localisation to the right iliac fossa. Our patient was pyrexial and had raised inflammatory markers, which thus mimicked acute appendicitis.

Depending upon the extent of the perforation and its anatomical site, as well as on the basis of the presence of diffuse or localised peritonitis, the treatment of foreign-body perforation will vary from simple suturing, with or without a protective colostomy, to exteriorisation in the form of a colostomy, and the Hartmann operation [11]. Our patient had a small perforation with minimal localised purulent peritoneal contamination and thus simple suturing of the perforation was performed. Consent was taken pre-operatively for appendicectomy only, so patients need to be made aware of all possible operative alternatives when there is a diagnostic dilemma.

## Conclusion

Diagnosis of an intestinal perforation can be difficult and requires a high degree of suspicion and awareness on the part of the clinician. Particularly, it can be challenging for a trainee surgeon who is most likely to be involved in operating on patients with suspected acute appendicitis. Our case emphasises an increased awareness of this diagnostic possibility, taking into consideration due implications on obtaining full consent from the patients; this is particularly important when there is a diagnostic dilemma, as this may have future medico-legal implications. It is important to inform patients about an alternative diagnosis and the very occasional need for bowel resection and stoma formation. Previous case reports have shown foreign-body perforations occurring through pathologically abnormal colons (abnormal due to carcinoma or diverticulum) [7-9]. In our patient, the sigmoid colon did not have any abnormal pathology.

## Consent

Written informed consent was obtained from the patient for publication of this case report and any accompanying images. A copy of the written consent is available for review by the Editor-in-Chief of this journal.

## Competing interests

The authors declare that they have no competing interests.

## Authors' contributions

SJ analyzed the patient's data, conceptualised the study and drafted the manuscript, IR performed the surgical procedure and helped in the literature search. SK helped in the literature search and obtained photos and images. SD supervised the process and finally approved manuscript for publication. All authors have read and approved the final manuscript.
